# Fungal Gene Mutation Analysis Elucidating Photoselective Enhancement of UV-C Disinfection Efficiency Toward Spoilage Agents on Fruit Surface

**DOI:** 10.3389/fmicb.2018.01141

**Published:** 2018-06-12

**Authors:** Pinkuan Zhu, Qianwen Li, Sepideh M. Azad, Yu Qi, Yiwen Wang, Yina Jiang, Ling Xu

**Affiliations:** School of Life Sciences, East China Normal University, Shanghai, China

**Keywords:** ultraviolet-C, fungal pathogen, photoreceptor, postharvest decay, *Botrytis cinerea*

## Abstract

Short-wave ultraviolet (UV-C) treatment represents a potent, clean and safe substitute to chemical sanitizers for fresh fruit preservation. However, the dosage requirement for microbial disinfection may have negative effects on fruit quality. In this study, UV-C was found to be more efficient in killing spores of *Botrytis cinerea* in dark and red light conditions when compared to white and blue light. Loss of the blue light receptor gene *Bcwcl1*, a homolog of *wc-1* in *Neurospora crassa*, led to hypersensitivity to UV-C in all light conditions tested. The expression of *Bcuve1* and *Bcphr1*, which encode UV-damage endonuclease and photolyase, respectively, were strongly induced by white and blue light in a *Bcwcl1*-dependent manner. Gene mutation analyses of *Bcuve1* and *Bcphr1* indicated that they synergistically contribute to survival after UV-C treatment. *In vivo* assays showed that UV-C (1.0 kJ/m^2^) abolished decay in drop-inoculated fruit only if the UV-C treatment was followed by a dark period or red light, while in contrast, typical decay appeared on UV-C irradiated fruits exposed to white or blue light. In summary, blue light enhances UV-C resistance in *B. cinerea* by inducing expression of the UV damage repair-related enzymes, while the efficiency of UV-C application for fruit surface disinfection can be enhanced in dark or red light conditions; these principles seem to be well conserved among postharvest fungal pathogens.

## Introduction

Fresh fruits and vegetables are rich in moisture and nutrients, and thus susceptible to postharvest decays caused by microbial contamination and proliferation, especially pathogenic fungi (Sperber et al., 2009). Chemical sanitizers are commonly used for disinfection of the harvested crops. However, the long-term use of chemical fungicides frequently poses the risk of fungicide resistance in pathogens. More importantly, pesticide residue in fresh crops is an increasing health concern among consumers. To address these issues, developing alternative methods to synthetic fungicides for disease management purpose is in urgent need (Romanazzi et al., 2012, 2016).

Ultraviolet-C (UV-C, 200–280 nm) offers interesting possibilities for postharvest disease management as a safe alternative to conventional chemical fungicides. Although the UV-C portion of the cosmic rays is almost completely absorbed by the outer space atmosphere and is hardly observed in nature on the earth’s surface, UV-C radiation can be created by artificial lamps, and usually causes two distinct effects on fresh fruits and vegetables: one is the elicitation of disease resistance and quality improvement in fresh crops, while the other is reduction of microbial population due to its direct germicidal effect (Urban et al., 2016). The former effect on host crops is often defined as hormesis, that is, stimulation of favorable responses in plants exposed to low or sublethal doses of an agent such as a physical stressor (Luckey, 1982). It has been recognized that UV-C light at low hormetic doses reduced the postharvest decay of a wide range of crops (Luckey, 1982), although these beneficial effects depend on the dose and timing of UV-C exposure, the fruit or vegetable species and cultivars, and the exposed area (Allende and Artés, 2003; Vicente et al., 2005; Costa et al., 2006; Pombo et al., 2011; Topcu et al., 2015). UV-C can cause DNA damage, and is thus used as a sterilizing agent for air, water and food (Bintsis et al., 2000). However, the host hormesis-inducing and microbe-disinfecting effects of UV-C on fresh crops are somewhat incompatible: the disease resistance elicitation effect can be achieved only when the UV-C dosage is restricted to certain sublethal dosages, while the microbe-disinfecting effect can be produced by increasing UV-C dosage. Accordingly, UV-C treatment of fruits and vegetables needs to be optimized tactically to obtain a desirable balance between the beneficial changes in host plants along with efficient disinfection against pathogens (Urban et al., 2016). To address this issue, it is important to understand the regulation mechanisms of UV-C resistance in fungal pathogens, which still remains to be elucidated.

It is known that UV-C inhibits microbial growth mainly by inducing the formation of pyrimidine dimers that alter the DNA helix and block microbial cell replication (Bintsis et al., 2000). However, microorganisms can protect themselves against UV radiation by repairing damaged DNA (Sinha and Hader, 2002). Proteins such as DNA photolyases have been found in a variety of species and can restore the UV-damaged bases back to their original undamaged states (Bluhm and Dunkle, 2008; Brettel and Byrdin, 2010). Additionally, the UV-damage endonuclease (UVDE) can directly recognize and cleave damaged DNA, which is followed by lesion removal, gap-filling, and ligation reactions (Bowman et al., 1994; Freyer et al., 1995; Yajima et al., 1995). Therefore, the UV-C dosages for fungicidal purposes must be relatively high, usually ranging from 0.5 to 20 kJ/m^2^ (Bintsis et al., 2000).

Fungi can also sense visible light to promote tolerance against harmful UV radiation (Fuller et al., 2015). This has been validated in several fungi by functional studies on the orthologs of White collar complex (WCC), the blue light receptor of *Neurospora crassa*. These proteins can act both as photosensors as well as transcription factors to regulate the expression of light responsive genes (Ballario et al., 1996; Crosthwaite et al., 1997; Liu et al., 2003). DNA repair enzymes, including photolyases and UVDE, were shown to be induced by light via the conserved WCC signaling pathway in several fungal species, including *Cryptococcus neoformans, Phycomyces blakesleeanus*, and *Ustilago maydis* (Verma and Idnurm, 2013; Tagua et al., 2015; Brych et al., 2016). The light regulation of UV-C resistance in fungi implies that ultraviolet disinfection efficiency can be adjusted by orchestrating photic conditions.

*Botrytis cinerea* is the gray mold pathogen that causes enormous economic damage to fruits and vegetables, both in field and during postharvest procedures (Fillinger and Elad, 2016). The infection cycle of this pathogen usually starts with the attachment of conidia to the plant surface, followed by infection and rapid hyphal spreading inside the plant tissue leading to host collapse (Fillinger and Elad, 2016). *B. cinerea* shows varied developmental responses to different wavelengths of the light spectrum. The conserved WCC homologs of *B. cinerea* mediate transcriptional responses to the blue light spectrum and inhibit its conidiation. Furthermore, WCC is required for coping with excessive light, oxidative stress, and to achieve full virulence to host plants (Canessa et al., 2013). Recently, cryptochrome/photolyase homologs, BcCRY1 and BcCRY2, were characterized in *B. cinerea*, revealing that BcCRY1 acts as the major photolyase in photoprotection, whereas BcCRY2 acts as a cryptochrome with signaling function in regulating repression of conidiation (Cohrs and Schumacher, 2017). However, the mechanism of photoselective regulation of UV resistance in *B. cinerea* has not been fully elucidated yet.

The present study aims to reveal the mechanism of regulation of UV-C resistance in the fungal fruit spoilage agents. Using *B. cinerea* as a representative model, we find that blue light and *Bcwcl1* are required for activating the expression of the UV-damage endonuclease and photolyase genes, *Bcuve1* and *Bcphr1* (or *Bccry1* in an earlier report, Cohrs and Schumacher, 2017), respectively. Gene mutation analysis revealed that *Bcuve1* and *Bcphr1* are synergistically responsible for coping with UV-C induced damage in *B. cinerea*. More importantly, since blue light is the specific spectrum that supports DNA-damage repair activities in fungi, UV-C treatment followed by dark or red-light conditions was thus found to enhance microbe-killing efficiency thereby facilitating fruit spoilage management.

## Materials and Methods

### Fungal Strains and Culture

The reference strain B05.10 of *Botrytis cinerea* was designated as wild type for genetic modification. The other pathogenic fungi were originally isolated from fruits and vegetables (**Table 1**). Potato dextrose agar (PDA) was used to maintain the fungal cultures at their indicated optimum temperatures. Conidia of each fungal species were collected by flooding the sporulated colonies with sterilized water, followed by filtration through four layers of cheesecloth and centrifugation at 4500 rpm. The concentration of the resulting conidial suspension was measured by a hematocytometer.

**Table 1:** Fungal species and growth temperature.

Species	Host origin	Temperature
*Botrytis cinerea*	Grape	25°C
*Alternaria alternata*	Broccoli	25°C
*Colletotrichum gloeosporioides*	Mango	28°C
*Penicillium digitatum*	Navel orange	25°C

### Visible and UV-C Light Treatment

White, blue, and red visible light spectra were produced by a light-emitting diode (LED, Qiding Photo Electronic, Shanghai, China). The parameters of each light spectrum are listed in **Table 2**. Light intensities were fixed at 20 μmol m^-2^s^-1^, as measured by Quantum Light Meter (Spectrum Technologies, United States), and achieved by manually adjusting the power control switch of the LED devices. For dark treatment, samples were kept in a light-proof plastic box and incubated at a temperature similar to the light-treated ones. HL-2000 cross-linker lamps (UVP, United States) were used for UV-C radiation (254 nm) treatment. The UV-C dosage was recorded as either μJ/cm^2^ or kJ/m^2^.

**Table 2:** Parameters of light.

Light color	Peak λ	Dominant λ	Average λ	Δλ peak
White	460.0	468.5	544.0	27.0
Blue	470.0	472.4	470.0	25.9
Red	675.0	663.3	674.0	24.3

*λ, wavelength (nm).*

### Generation of Gene Deletion Mutant

Protoplast transformation mediated homologous recombination strategy was adopted to generate knock out mutants of target genes according to the previous method (Chung and Lee, 2015). 1 kb of the 5′ and 3′ untranslated regions (UTRs) of target genes were amplified from the DNA of the wild type strain, and selective marker genes, hygromycin (*hyg*) or nourseothricin resistant (*nat*) cassette, were PCR amplified from the plasmid pCAMBI1300 or pNR2, respectively. Overlap PCR was then performed to fuse the 5′- and 3′-UTRs with the selective marker genes, resulting in 5′ UTR-*hyg* (or *nat*) -3′ UTR constructs for protoplast transformation. Diagnostic PCR was performed to identify bonafide targeted disruption mutants among the emerged transformants as indicated in **Supplementary Figure S1**. The gene disruption mutants were further verified by Southern blot hybridization according to the protocol recommended in the DIG high prime DNA labeling and detection starter Kit II (Roche, Mannheim, Germany).

### Construction of *Bcuve1-GFP* Fusion Expression Strain and Fluorescent Microscopy

The expression vector pNDN-OGG (Schumacher, 2012) carrying nourseothricin resistance (*NAT*) and *GFP* expression cassettes was digested with *Nco*I. The wild type *Bcuve1* was amplified without the stop codon using the primers P45/46 (**Supplementary Table S1**) that are equipped with 22-bp overlaps corresponding to the sequences in the destination vector for the Gibson assembly-based cloning using the Hieff Clone^TM^ Plus Multi One Step Cloning Kit (YEASEN, China). The resulted clones were identified by PCR diagnosis, and the positive ones were further confirmed for correctness by sequencing. The correct vector named pNDN-OGG-*Bcuve1* carrying *Bcuve1* upstream of the *GFP* gene was linearized by SacII digestion and transformed into Δ*bcuve1.* The fungal transformation was selected by nourseothricin (50 μg/ml). The positive resistant transformants were purified by series of single spore culture, and the Δ*bcuve1*-*Bcuve1*-*GFP* strain was obtained for UV-C sensitivity and microscopy analysis. Fluorescence and light microscopy was performed with a Zeiss Axio Imager Z2 microscope. Differential interference microscopy (DIC) was used for bright field images. GFP fluorescence was examined using excitation BP 470/40 and emission BP 525/50, DAPI staining with the excitation G 365 and emission BP 445/50. DIC, GFP, and DAPI images were merged via ImageJ soft ware.

### Gene Expression Analysis

Aliquots (200 μl) of conidial suspensions (10^6^ conidia/ml) were inoculated on cellophane-overlaid PDA and incubated at 25°C in dark for 24 h. Samples were subsequently divided into four groups each and placed under different light conditions (white, blue, red light, and darkness). After incubating for 1 h, mycelium samples (about 0.1 g) from each of the groups were harvested using cell scrapers in the dark, transferred into 2 ml Eppendorf tubes, and immediately frozen in liquid nitrogen. For total RNA extraction, each sample was submerged in 1 ml Trizol reagent (Invitrogen, Carlsbad, CA, United States), and homogenized by shaking along with four steel balls (2 mm diameter) at 70 Hz and 4°C for 3 min on a Tissuelyser (Jingxin Industrial, Shanghai, China). The resulting suspensions were extracted with chloroform according to the manufacturer’s instructions supplied with Trizol. One microgram of each RNA sample was used as a template for reverse transcription using the Prime Script^TM^ RT reagent Kit (Perfect Real Time) (TakaRa Biotechnology, Co., Dalian, China). Real-time PCR amplifications were conducted in a CFX96^TM^ Real-Time System (BIO-BAD, Inc., United States) using TakaRa SYBR Premix Ex Taq (TakaRa Biotechnology). Relative quantifications of the real-time PCR amplifications were performed with the following parameters, initial preheating at 95°C for 30 s followed by 39 cycles at 95°C for 5 s and 60°C for 30 s. The β-tubulin gene was analyzed as an internal reference. Experiments were repeated three times for each sample. The primers used in this study are listed in **Supplementary Table S1**. The gene expression levels were calculated using the 2^-ΔΔCt^ method (Livak and Schmittgen, 2001). All experiments were repeated three times.

### UV-C Sensitivity Assays

To evaluate fungal UV-C sensitivity, 200 μl of conidial suspensions (5 × 10^3^ conidia/ml) were evenly inoculated on individual PDA surfaces using a cell spreader and subjected to UV-C irradiation, with dosages ranging from 0.6 to 1.2 kJ/m^2^. The samples were immediately incubated at 25°C for 2 h under white, blue, red light and dark conditions. Subsequently, all samples were continuously incubated in the dark for 2 days. Samples that were not subjected to UV-C treatment were used as reference controls. Fungal colonies arising on the plate were counted and survival rates were calculated by dividing the colony numbers on UV-C treated plates with those on non-UV-C treated ones.

To visualize the effect of visible light on fungal UV-C sensitivity, 5 μl of conidial suspension (10^6^ conidia/ml) was dropped on cellophane overlaid with water agar. After UV-C radiation, the samples were similarly treated for 2 h under different light and dark conditions, and then incubated in the dark for another 22 h. Conidial germination of each sample was finally examined under a light microscope. Five replicates were conducted for all experiments.

### Fruit Inoculation Assay

Wild type spores of *B. cinerea* were suspended in sterilized 1% sugar solution, and the concentration was adjusted to 10^6^ conidia/ml. Table grapes were purchased from the local super market. Healthy berries with uniform size and maturity stage were selected for this assay. Before inoculation, the fruits were submerged in 0.5% sodium hypochlorite solution for 3 min to eliminate possible contaminating microorganisms on the surface of grape berries, followed by rinsing thrice with sterilized water. The fruits were then artificially wound-inoculated with 10 μl spore suspension at a site on the equatorial line of each grape berry. The fruits were then exposed to 1 kJ/m^2^ UV-C, and divided into four groups, each including 30 berries, and transferred to dark, white, blue, and red lights to incubate at 25°C for 2 h. Subsequently, all samples were placed in continuous dark, and 4 days later the disease symptoms were photographed, and the decay areas were measured via ImageJ software.

### Statistical Analysis

The experiments in this study were repeated three times. The data obtained were analyzed by ANOVA followed by Duncan’s multiple range tests (*p* < 0.01) for means comparison with the use of SPSS 17.0.

## Results

### Blue Light Is Specifically Required for Inducing UV-C Resistance in a *Bcwc1*-Dependent Manner

**FIGURE 1:**
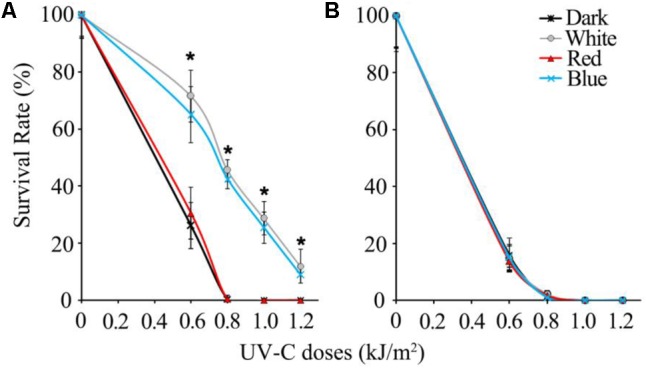
Survival rates of UV-C treated spores of wild type **(A)** and *Δbcwcl1* **(B)** strains of *B. cinerea*. Error bars represent standard deviation. Asterisks (^∗^) indicate that survival of the spores illuminated by blue and white light after UV-C stress was significantly higher than those treated by red light and dark (*P* < 0.01). Error bars represent SD and *n* = 5.

In the UV-C sensitivity assay, wild type spores of *B. cinerea* were completely killed by 0.8 kJ/m^2^ UV-C in dark and 2 h red light-treated groups, while the spores exposed to white and blue light survived by more than 40%. Even when the UV-C dose reached 1.2 kJ/m^2^, the wild type spores illuminated in white and blue light maintained an approximately 10% survival rate (**Figure 1A**). Eventually, we confirmed that the blue light spectrum (but not the red light) is specifically effective in enhancing *B. cinerea* spore survivability after UV-C irradiation.

Since blue light is known to be sensed by fungi via the WCC photoreceptors, the WC-1 homolog gene in *B. cinerea, Bcwcl1* (Canessa et al., 2013), was disrupted by replacing the open reading frame with the hygromycin resistance cassette via homologous recombination. *Δbcwcl1* mutant strains were confirmed by genomic PCR, and showed enhanced melanization and sporulation in contrast to the wild type strain (data not shown). These phenotypes were in agreement with a previous report in which WC-1 was shown to negatively regulate spore formation and melanin biosynthesis (Canessa et al., 2013). One representative *Δbcwcl1* strain was thus used for the UV-C sensitivity assay. The results showed that neither blue nor white light could increase survivability of the mutant after UV-C radiation. The survival rates of the *Δbcwc1* mutant dropped down to almost 0% in all the treatment groups when the UV-C dosage was above 0.8 kJ/m^2^ (**Figure 1B**). Taken together, we conclude that photoinduction of UV-C resistance in *B. cinerea* is specifically caused by the blue light spectrum via signaling through the light receptor encoded by *Bcwcl1.*

### Expression of DNA Damage Repair Related Genes Is Regulated by Light via *Bcwcl1*

**FIGURE 2:**
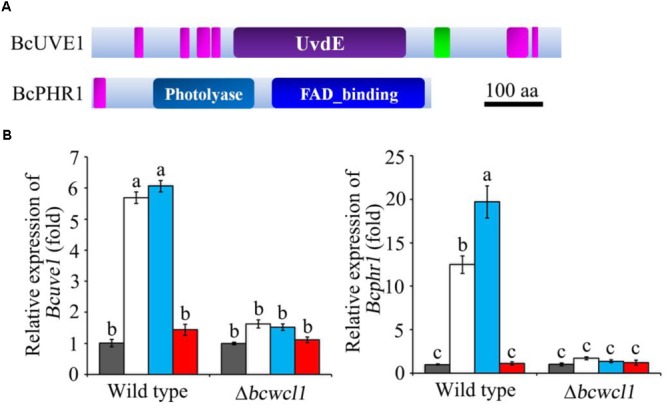
Expression analysis of DNA damage repair related genes. **(A)** The Ensembl Fungi IDs of *Bcuve1* and *Bcphr1* are Bcin01g08960.1 and Bcin05g08060.1, respectively. According to SMART analysis, the BcUVE1 protein contains a UV damage endonuclease domain (UvdE), and BcPHR1 contains a DNA_photolyase domain and a FAD_binding domain. The pink rectangles represent regions of low complexity, and the green one represents the coiled-coil region. The scale bar indicates a length of 100 amino acids. **(B)** Comparison of *Bcuve1* and *Bcphr1* expression levels between the wild type and *Δbcwcl1* strains under different light conditions. Columns represent dark, white, blue, and red light exposure from left to right. Histograms marked with different characters are statistically different (*p* < 0.01). Error bars represent SD and *n* = 3.

Since white collar 1 can function as both blue light receptor and transcription factor (Canessa et al., 2013), it is assumed that certain downstream genes regulated by this protein could be responsible for photo responsive phenotypes. Based on the transcriptomic data (Schumacher et al., 2014), two genes expected to contribute to DNA damage repair in *B. cinerea* were obtained: *Bcuve1* (Bcin01g08960) encoding the protein homologous to the UV damage endonuclease Uve1 of *Schizosaccharomyces pombe* (GenBank: CAA19577.1), and *Bcphr1* (Bcin05g08060, or *Bccry1* in Cohrs and Schumacher, 2017) encoding the homolog of photolyase/cryptochrome of *Neurospora crassa* (GenBank: KHE81232.1). The deduced protein domains of these two gene products are presented in **Figure 2A**. The expression of *Bcuve1* and *Bcphr1* was analyzed via quantitative RT-PCR. The results showed that white and blue light treatments strongly induced the expression of *Bcuve1* and *Bcphr1* in the wild type, but not in the *Δbcwcl1* mutant strain. However, red light exposure did not change the expression of these two genes in both WT and *Δbcwcl1* strains (**Figure 2B**). Taken together, these results indicate that blue light signaling via the WC-1 homolog activates both endonuclease excision and photolyase pathways in *B. cinerea*.

### *Bcuve1* and *Bcphr1* Synergistically Contribute to UV-C Resistance in *B. cinerea*

**FIGURE 3:**
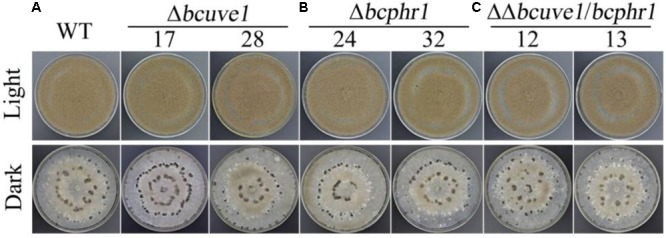
The three mutant strains, **(A)** *Δbcuve1*, **(B)** *Δbcphr1*, and **(C)** *ΔΔbcuve1/bcphr1*, formed similar sporulation colonies (1 week in light) and sclerotia (2 weeks in dark) on PDA.

**FIGURE 4:**
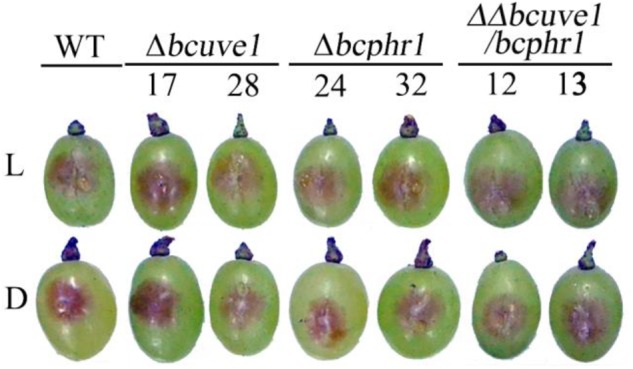
Bcuve1 and Bcphr1 are not required for virulence. Grape berries were inoculated with spore suspensions of each strain, and disease development was checked after 3 days of incubation at 22°C in light (L) or dark (D).

To confirm the roles of *Bcuve1* and *Bcphr1* in *B. cinerea*, single and double mutant strains were created via protoplast transformation and homologous recombination mediated gene replacement. Genomic PCR analysis and Southern blot confirmed mutation of the targeted locus in the colonies recovered (**Supplementary Figure S1**). The resulting mutants, *Δbcuve1, Δbcphr1*, and *ΔΔbcuve1/bcphr1*, showed growth rates, sporulation, sclerotial development (**Figure 3** and **Table 3**), and virulence equivalent to the wild type strain when tested on grape berries (**Figure 4**), indicating that neither *Bcuve1* nor *Bcphr1* is involved in regulating the vegetative growth, development, and host-invasion processes.

**Table 3:** Comparison of colony growth, sporulation, and sclerotia formation.

Genotypes of fungal strains	Growth rate (mm/day)		Sporulation	Sclerotia
	Dark	Light	(× 10^7^/dish)	(No./dish)
WT	28.57 ± 0.2	28.48 ± 0.17	5.1 ± 0.15	167.6 ± 5.24
*Δbcuve1*-17	28.16 ± 0.18	28.36 ± 0.21	4.67 ± 0.13	163.83 ± 4.06
*Δbcuve1*-28	28.13 ± 0.31	28.06 ± 0.31	5.05 ± 0.11	61.83 ± 3.7
*Δbcphr1*-24	27.98 ± 0.37	28.16 ± 0.41	5.06 ± 0.12	162.6 ± 5.04
*Δbcphr1*-32	28.5 ± 0.23	28.45 ± 0.19	4.92 ± 0.14	163.4 ± 1.72
*Δbcuve1/bcphr1*-12	28.23 ± 0.46	28.89 ± 0.39	5.04 ± 0.11	161.6 ± 4.76
*Δbcuve1/bcphr1*-13	28.8 ± 0.27	27.95 ± 0.43	4.76 ± 0.1	164.5 ± 3.96

*Data in this table were measured from cultures grown in CM medium. Sporulation and sclerotia formation were tested with 1 week old cultures in constant light and 2 week old cultures in constant dark, respectively.*

**FIGURE 5:**
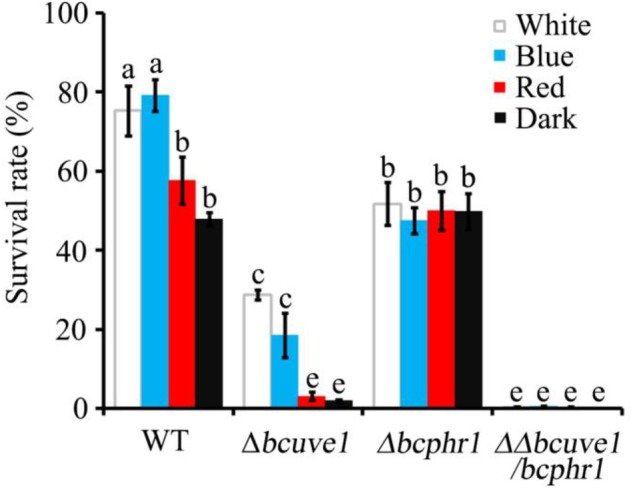
The three mutant strains, *Δbcuve1, Δbcphr1*, and *ΔΔbcuve1/bcphr1*, showed differentially altered sensitivities to UV-C (0.6 kJ/m^2^) when influenced with visible light qualities. Bars marked with non-identical characters are significantly different (*P* < 0.01). Error bars represent SD and *n* = 5.

In the UV-C sensitivity assay, the *Δbcuve1* mutant showed significantly reduced survival rates when compared to the wild type strain. However, blue and white lights were still capable of enhancing UV-C tolerance of the *Δbcuve1* mutant when the dosage was 0.6 kJ/m^2^ (**Figure 5**). On the other hand, the *Δbcphr1* mutant was relatively more tolerant to UV-C than *Δbcuve1*, although *Δbcphr1* still showed significantly reduced survival rate under UV-C stress when compared to the wild type. Visible light treatment after UV-C radiation did not alter the survivability of *Δbcphr1* (**Figure 5**). Moreover, the double mutant, *ΔΔbcuve1/bcphr1*, combined the patterns of the two single mutants, showing similar sensitivity to UV-C as the *Δbcuve1* mutant in dark, and no change in survival rate as the *Δbcphr1* mutant when treated with white light after UV-C treatment (**Figure 5**). These data together indicate that BcUVE1 and BcPHR1 play synergistic roles in UV-C damage repair in *B. cinerea*.

**FIGURE 6:**
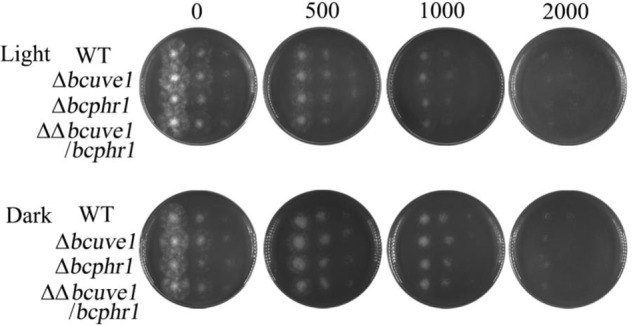
Sensitivity to hydrogen peroxide (H_2_O_2_). Spore suspensions of each tested strain were serially diluted to 10^6^, 10^5^, and 10^4^ spores per ml, and 10 μl of each suspension was spotted on PDA medium (vertical array of 4 spots for each diluted suspension). The H_2_O_2_ contents (0–2000 mM) in the media are indicated for each column. The plates were photographed after 2 days of incubation at 22°C in light (L) or dark (D).

Since UV radiation can stimulate organisms to generate reactive oxygen species (ROS), which can also cause DNA damage, we additionally tested the sensitivity of each strain to ROS stress upon treatment with hydrogen peroxide (H_2_O_2_). The results demonstrated that all the strains, i.e., *Δbcuve1, Δbcphr1, ΔΔbcuve1/bcphr1* and WT, showed decreased survivability with increasing H_2_O_2_ concentration in the medium, however, no significant difference in susceptibility to H_2_O_2_ was observed between the mutants and wild type (**Figure 6**).

### Role of BcUVE1 in UV-Damage Repair Is Confirmed by Genetic Complementation and Subcellular Localization

**FIGURE 7:**
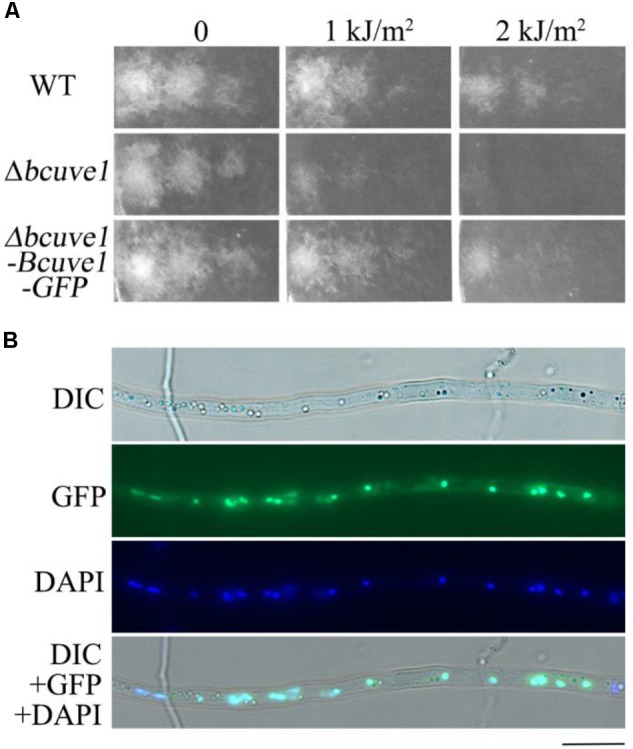
Mycelial development of test fungal strains exposed to UV-C. **(A)** *Bcuve1*-*GFP* complementation to detect UV-sensitivity of the Δ*bcuve1* mutant. **(B)** Location of the fused marker protein in the nuclei of the Δ*bcuve1*-*Bcuve1*-*GFP* mutant as shown in the light (upper) and fluorescent (lower) microscopes.

In order to confirm that the UV-tolerance deficiency of *Δbcuve1* mutant is due to disruption of the *Bcuve1* gene, wild type *Bcuve1* was tagged with *GFP* at the 3′ end and transformed into Δ*bcuve1* to produce the complementation mutant strain *Δbcuve1*-*Bcuve1*-*GFP*. UV sensitivity assays showed that survival of *Δbcuve1*-*Bcuve1*-*GFP* was similar to the WT (**Figure 7A**). Since the UV-endonuclease is supposed to be involved in DNA damage repair, we determined the subcellular localization patterns of BcUVE1 by tracking the constitutively expressing GFP fusion proteins in the *Δbcuve1*-*Bcuve1*-*GFP* strain. As expected, the fused protein BcUVE1-GFP was found in the nuclei (**Figure 7B**).

**FIGURE 8:**
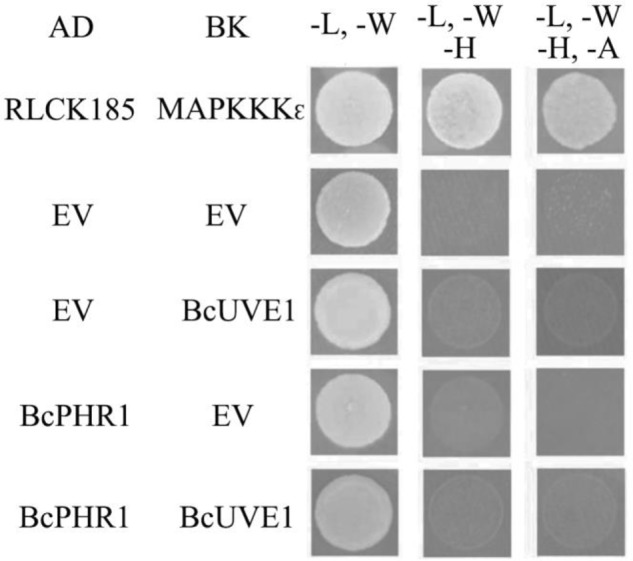
Yeast two hybrid assays: BcUVE1 and BcPHR1 do not interact with each. The cDNAs of either gene were cloned adjacent to the activation (AD) or DNA binding (BK) domains of *S. cerevisiae* Gal4. Constructs were transformed into the *S. cerevisiae* reporter strain AH109. Growth on medium without leucine (L), tryptophan (W), histidine (H), and adenine (A) indicate interactions between tested alleles to reconstitute the Gal4 protein. AD-RLCK185 and BK-MAPKKK_𝜖_ are positive controls that have been proved to physically interact with each other (Wang et al., 2017).

Additionally, yeast two hybrid experiments indicated that BcUVE1 and BcPHR1 do not interact with each other (**Figure 8**), even though BcPHR1 (or named as BcCRY1 earlier) is also localized in nuclei (Cohrs and Schumacher, 2017), and both BcUVE1 and BcPHR1 are involved in UV-damage repair.

### Fungicidal Efficiency of UV-C on Fruits Is Enhanced in Red Light and Dark Conditions but Not in Blue or White Light Conditions

**FIGURE 9:**
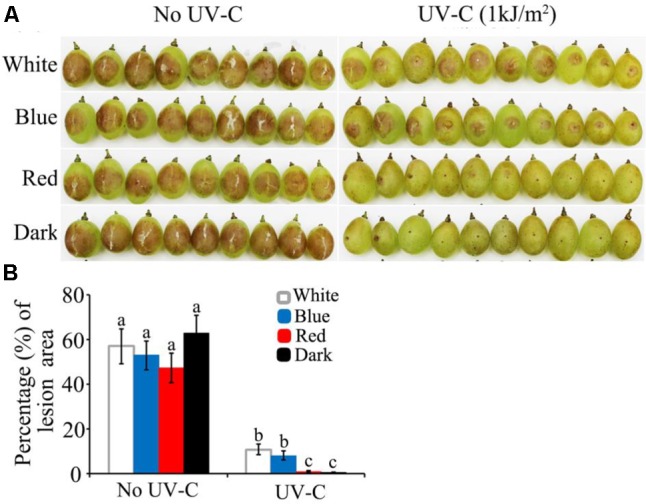
UV-C radiation achieved more efficient restriction of grape decay in red light and dark than in blue and white light conditions. **(A)** Photograph records of the infected grape berries on 4th day after inoculation. **(B)** Data analysis for the percentage of decayed area on each grape berry. White, blue, red, and dark represent light quality treatment. The data followed by different characters are statistically different (*p* < 0.01).

The above study demonstrated that *B. cinerea* is more susceptible to UV-C stress in dark and red light than in blue and white light. These findings enabled us to make an association between the process of light-regulated UV damage repair mechanism and UV-C application for plant disease control, especially at the postharvest stage. The present assay indicated that artificial inoculation of wild type *B. cinerea* spores on grape berries would fail to cause decay symptoms if the UV-C (1 kJ/m^2^) treatment was followed by dark or red light (**Figure 9**). In contrast, exposure to blue and white light caused the UV-C treated samples to finally develop typical soft decay (**Figure 9**). Consequently, the *in vitro* and *in vivo* assays suggest that more satisfactory results of UV-C application for postharvest disease management can be expected if the UV-C damage repair activities of the fungal pathogens are suppressed.

### Visible Light Qualities Show Similar Effects on UV-C Sensitivity of Common Postharvest Fungal Pathogens

**FIGURE 10:**
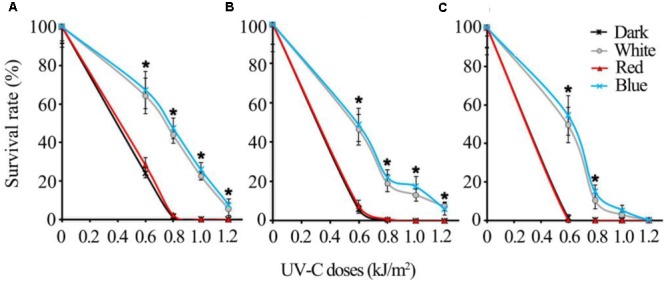
The UV-C sensitivity of three common postharvest fungal pathogens is regulated similarly by different visible light qualities **(A)** *Alternaria alternata*; **(B)** *Penicillium digitatum*; **(C)** *Colletotrichum gloeosporioides*. Asterisks (^∗^) indicate that survival of spores illuminated by blue and white light after UV-C stress was significantly higher than those treated by red light and dark (*P* < 0.01). Error bars represent SD and *n* = 5.

The regulatory mechanisms of UV-C resistance uncovered here are expected to be valid even in other pathogenic fungi. In this study, we additionally tested the UV-C sensitivity of other important postharvest fungal pathogens: *Alternaria alternata, Penicillium digitatum*, and *Colletotrichum gloeosporioides*. The results suggest that all of the fungi tested were killed much more easily by relatively lower dosages (less than 1 kJ/m^2^) of UV-C in red light and dark conditions, while the spores exposed to blue and white light could survive from higher UV-C dosages (**Figure 10**).

## Discussion

Spoilage decay due to contamination by pathogenic fungi is one of the main causes of abundant postharvest losses of fresh fruits and vegetables. UV-C is an alternative to fungicides for control of postharvest diseases, due to its dual roles of inducing defense in plants and causing surface disinfection of the pathogenic microbes. Induced resistance to postharvest pathogens by UV-C was shown in a wide range of crops (Ben-Yehoshua et al., 1992; Mercier et al., 1993; Charles et al., 2008a,b). However, decontamination of the fruit surface by UV-C could still be interesting from a practical standpoint, as the irradiated tissues would be subject to less inoculum pressure in addition to being more disease resistant. Thus, the present work has been focused on the pathogen rather than the host.

From an evolutionary perspective, the presence of light may signal the upcoming threat of genotoxic ultraviolet radiation to microbes in the natural environment and thus activate UV-damage repair activities (Fuller et al., 2015). Therefore, our study attempted to address the knowledge gap of light-regulation mechanisms of UV-C tolerance in phytopathogenic fungi, and lay the foundation to optimize UV-C treatment parameters for better disinfection efficiency on fresh crop surfaces.

Through quantitative UV-C sensitivity assays with the model species *B. cinerea*, we found that the spores of this fungus incubated in red light and dark are more sensitive to UV-C than those incubated in white and blue light conditions. This phenomenon implies that the blue light spectrum is capable of inducing UV-C resistance in *B. cinerea*. Since the WCC proteins are known to be conserved blue light receptors in the fungal kingdom, we further investigated the role of the key component of the WCC, BcWCL1 of *B. cinerea*, in UV-C resistance. A *Bcwcl1* deletion mutant showed substantially reduced UV-C resistance under any light conditions, confirming that the blue light receptor system of *B. cinerea* indeed regulates UV-C resistance. This is in accordance with the reports that WC-1 homologs are pivotal for environmental UV stress tolerance in several other fungal species (Idnurm and Heitman, 2005; Ruiz-Roldan et al., 2008; Kim et al., 2014).

The photoreceptor WCC can serve both signal input (LOV domain) and output (Zn-finger transcription factor domain) functions. Photo induction of DNA repair enzymes represents one of the downstream signaling targets of WCC (Fuller et al., 2015). Photoreactivating enzymes such as photolyases are induced by light via WCC homologues in the ascomycetes *Neurospora crassa, Aspergillus fumigatus* (Fuller et al., 2013), *Aspergillus nidulans* (Ruger-Herreros et al., 2011), *Fusarium oxysporum*, (Ruiz-Roldan et al., 2008) and *Cercospora zeae-maydis* (Bluhm and Dunkle, 2008; Kim et al., 2011), as well in as the basidiomycete *Ustilago maydis* (Brych et al., 2016). In these fungi, photolyases are recognized as the major enzymes responsible for UV damage repair. Visible light likely plays dual roles in enhancing photolyase-dependent UV resistance in fungi, one being the induction of expression of photolyase genes via WCC signaling, while the other being energy provision to support photoreactivation activity of the photolyases (Fuller et al., 2015). In *B. cinerea*, there are two cryptochome/photolyase homologs, BcCRY1 and BcCRY2, but only BcCRY1 was found to act as the major photolyase in photoprotection (Cohrs and Schumacher, 2017), which we therefore re-named as BcPHR1. We confirmed that the expression of *Bcphr1* was induced by white and blue light in a *Bcwcl1*-dependent manner, and the deletion mutant *Δbcphr1* showed increased UV-sensitivity when compared with WT as measured by quantitative spore survivability assay. However, *Δbcphr1* was found to be relatively more resistant to UV-C than *Δbcwcl1*, implying that *Bcphr1* is not the only member of the WCC downstream targets responsible for UV-damage repair.

Actually, as shown in the light-induced transcriptome data (Schumacher et al., 2014), *Bcuve1* represents another candidate for repairing UV- induced damages. This gene encodes a protein that is homologous to UVDE in fission yeast *Schizosaccharomyces pombe*, in which UVDE is essential for excision repair of UV induced DNA damage (Bowman et al., 1994; Freyer et al., 1995; Yajima et al., 1995). Additionally, Uve1, the UVDE homolog in the basidiomycetes *C. neoformans*, is a direct target of WCC signaling and required for UV resistance (Verma and Idnurm, 2013). As shown in our study, expression of *Bcuve1* is also enhanced by blue and white light in a *Bcwcl1*-dependent manner, and the deletion mutant *Δbcuve1* is more sensitive to UV-C than WT. However, white and blue light still moderately elevated survival rate of *Δbcuve1* spores after UV-C treatment, which is most probably due to the presence of functional *Bcphr1* in this mutant. This hypothesis was confirmed by analysis of the double mutant *ΔΔbcuve1/bcphr1*, which showed almost similar deficiency of UV-C tolerance as the *Δbcwcl1* mutant to any kind of light conditions. Taken together, WCC mediated blue light signaling in *B. cinerea* can activate both UV-endonuclease and photolyase to synergistically repair damages caused by UV-C radiation.

The major damage caused by UV-C to organisms is DNA lesions. Thus, subcellular localization of each DNA damage repair enzyme is indicative of its functional preference on either the nuclear or cytoplasmic (the mitochondrion) genomes. BcCRY1 (or BcPHR1 in this paper) was shown to solely localize in the nuclei (Cohrs and Schumacher, 2017). Interestingly, this study found that the UV-endonuclease BcUVE1 also accumulated in the nuclei as shown by analysis of the GFP tagged allele. Thus, these two DNA damage repairing enzymes are regulated by blue light signaling in *B. cinerea*, and are presumably responsible for removing UV-induced lesions in the nuclear genome. However, yeast-two-hybrid assays demonstrated that BcPHR1 and BcUVE1 did not interact with each other, further implying that these two enzymes mediated two independent DNA repair pathways. In addition, the spores of the *Δbcuve1* strain were more sensitive to UV-C than those of *Δbcphr1*. This phenotype could possibly be explained by the different DNA damage precursors they repair. It is well known that the major DNA lesions induced by UV-C are cyclobutane pyrimidine dimers (CPD) (Watanabe et al., 2006), and other minor lesions are pyrimidine pyrimidone photoproducts (6-4PP) and some diverse rare DNA photoproducts (Stapleton, 1992). BcPHR1 (or BcCRY1) is phylogentically recognized as belonging to the CPD photolyase group (Cohrs and Schumacher, 2017), and therefore its target precursors may be limited to CPD lesions. On the other hand, UV-endonuclease was originally discovered in *S. pombe* to be able to recognize both CPD and 6-4PP and initiate their excision repair (Bowman et al., 1994; Freyer et al., 1995; Yajima et al., 1995), even though CPD and 6-4PP differ significantly with respect to the structural distortions that they induce in the DNA duplex. The homolog of UV-endonuclease in *B. cinerea*, BcUVE1, is probably more versatile than the photolyase BcPHR1 in its DNA damage repair capability.

White collar complex of *B. cinerea* were found to be involved in tolerance to ROS (Canessa et al., 2013), which can also cause DNA damage and affect virulence. However, the test of sensitivity against hydrogen peroxide demonstrated that neither *Bcuve1* nor *Bcphr1* was involved in detoxification of ROS stress. Besides, the deletion mutants did not show any notable defects in vegetative growth, sporulation, sclerotial formation, or virulence, suggesting that BcUVE1 and BcPHR1 specifically cope with UV stress in *B. cinerea*.

As discussed earlier, UV-C could be used as a potential agent for sanitization of fresh fruit and vegetable surfaces (Nigro et al., 1998). However, the efficacy of UV-C is dependent on the resistance of target mircroorganisms against UV-C light (Syamaladevi et al., 2013). Based on our study, it can be deduced that the germicidal effect of UV-C on fungal pathogens can be attenuated by exposure to visible light, especially the blue light spectrum, largely due to induction of DNA damage repair enzymes (BcUVE1 and BcPHR1) by light. So, these findings may theoretically verify the rationality of an earlier practical study reporting that dark period following UV-C treatment enhances killing of *Botrytis cinerea* conidia and controls gray mold of strawberries better in green houses (Janisiewicz et al., 2016). Furthermore, we expanded this photoselective enhancement of UV-C disinfection into postharvest disease management. Consequently, UV-C application for postharvest disease management can be more effective if the UV-C damage repair activities of the fungal pathogens are suppressed by either dark or red light conditions. Additionally, the UV-C sensitivities of several common fungal pathogens behaved similarly under different light conditions. These phenomena can be explained by the fact that the WCC homologs are widely conserved blue light receptors in the fungal kingdom (Fuller et al., 2015), with the UV damage repair systems being one of their common regulation targets (Idnurm and Heitman, 2005; Verma and Idnurm, 2013; Schumacher et al., 2014; Wu et al., 2014; Brych et al., 2016). As a result, common postharvest pathogenic fungi can be efficiently killed by relatively less amounts of UV-C by following the principles stated in this study. Based on the phenomena and their underlying mechanisms discovered in this study, a shelf device equipped with UV-C inside and a red monochromatic filter on the screen is proposed to be beneficial for better disease management of fresh postharvest crops (**Supplementary Figure S2**).

Although UV-C is directly germicidal to microbial agents of postharvest diseases, its application for conservation purpose of fresh fruit and vegetables is also largely influenced by its effect on physiological modifications of the commodities. The possibility of injuries to crops by higher UV-C doses could even cause an increase in the susceptibility of fruits to postharvest decays (Stevens et al., 1996). We achieved enhancement of UV-C disinfection efficiency on the pathogen with limited dosages that are significantly less than those being commonly used to irradiate fresh crops. Subsequently, future efforts should be focused on selecting proper UV-C parameters to obtain beneficial effects without causing detrimental changes on quality attributes.

## Author Contributions

PZ and LX designed the experiments. QL, SA, YQ, YW, and PZ performed the experiments. QL, SA, YQ, PZ, YJ, and LX analyzed and interpreted the data. QL, SA, PZ, and LX wrote the paper with insight from all the authors.

## Conflict of Interest Statement

The authors declare that the research was conducted in the absence of any commercial or financial relationships that could be construed as a potential conflict of interest. The reviewer ZZ and handling Editor declared their shared affiliation.

## Supplementary Material

FIGURE S1 Strategies for deletion of *Bcuve1* and *Bcphr1.* **(A)** Schematic illustration of the homologous recombination strategy to replace the 5′ end of the target gene with hygromycin B (*hph*) or nourseothricin (*Nat*) resistance cassette as selective markers. **(B)** Diagnostic PCR analysis for integration of the replacement fragment with genomic DNA. As demonstrated in **(A)**, primer pairs P_TF_-P_TR_ and P_MF_-P_MR_ were used to test the presence or absence of target genes (*Bcuve1* or *Bcphr1*) and selection markers (*hph* or *Nat*), respectively; the primer pairs P_T5F_-P_MR′_ and P_MF′_-P_T3R_ were used to verify the correctness of integration sites at the 5′-UTR and 3′-UTR regions respectively. **(C)** Southern blot analysis of the WT, Δ*bcuve1* and *Δbcphr1* strains. Genomic DNAs were digested with *Hin*dIII; the probes targeting the selection markers are indicated in **(A)**. A single band of expected size in each mutant verified authentic homologous recombination events, and ruled out the possibility of multicopy insertion of the selection markers. **(D)** Reverse transcript-PCR confirmed the absence of expression of *Bcuve1* and *Bcphr1* in the respective mutants. The constitutively expressed gene *β-tubulin* was used as a reference.

FIGURE S2 Shelf device equipped with UV-C inside and a monochromatic filter on the screen for fresh crop preservation and display.

TABLE S1 Primers used in this study.
