# Supplementary figures and images for: Fungal Gene Mutation Analysis Elucidating Photoselective Enhancement of UV-C Disinfection Efficiency Toward Spoilage Agents on Fruit Surface
Source: Front Microbiol. 2018 Jun 12;9:1141. doi: 10.3389/fmicb.2018.01141 (PMC6008522; doi:10.3389/fmicb.2018.01141)

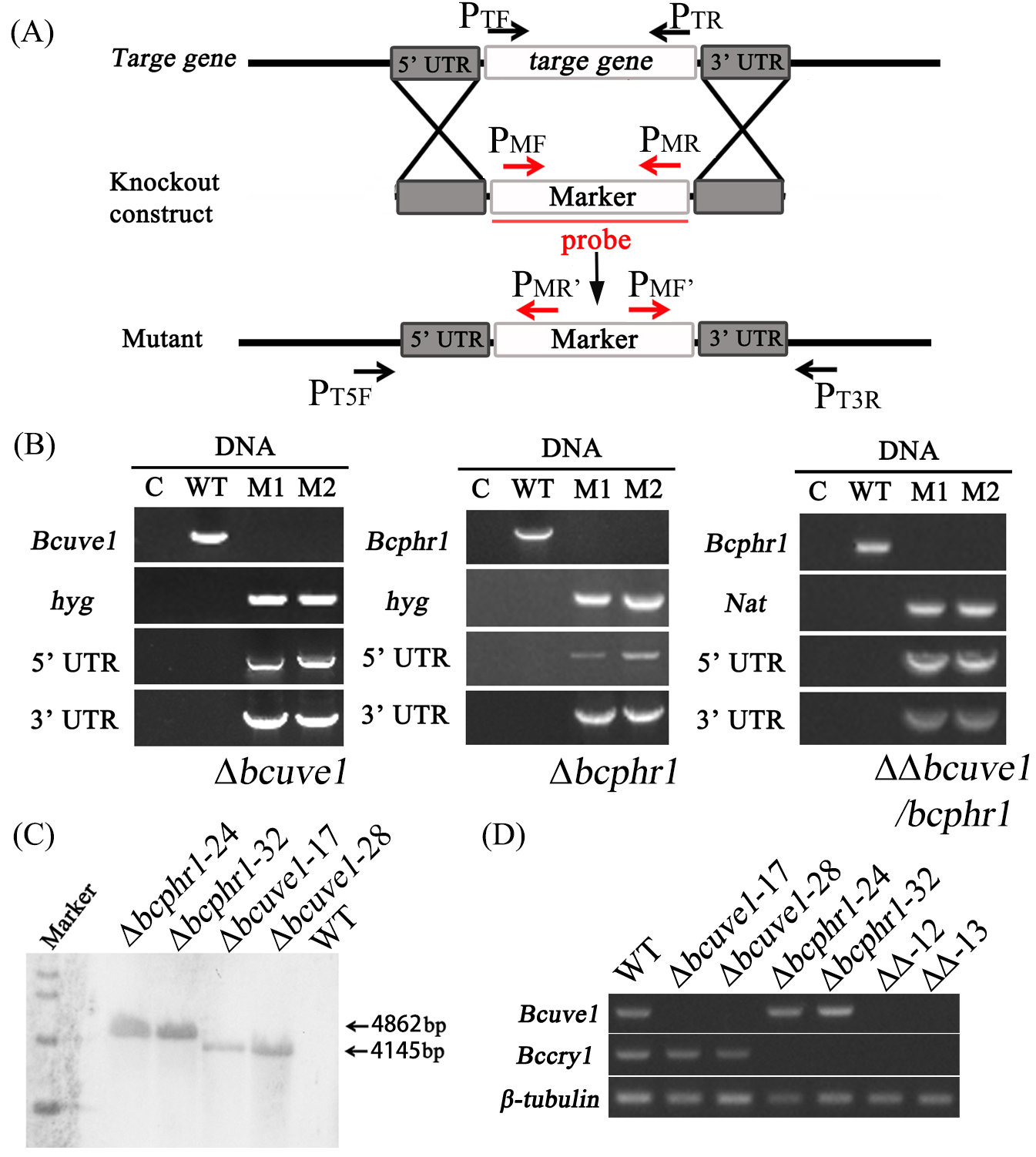

Supplement: FIGURE S1 — Strategies for deletion of Bcuve1 and Bcphr1. (A) Schematic illustration of the homologous recombination strategy to replace the 5′ end of the target gene with hygromycin B (hph) or nourseothricin (Nat) resistance cassette as selective markers. (B) Diagnostic PCR analysis for integration of the replacement fragment with genomic DNA. As demonstrated in (A), primer pairs PTF-PTR and PMF-PMR were used to test the presence or absence of target genes (Bcuve1 or Bcphr1) and selection markers (hph or Nat), respectively; the primer pairs PT5F-PMR′ and PMF′-PT3R were used to verify the correctness of integration sites at the 5′-UTR and 3′-UTR regions respectively. (C) Southern blot analysis of the WT, Δbcuve1 and Δbcphr1 strains. Genomic DNAs were digested with HindIII; the probes targeting the selection markers are indicated in (A). A single band of expected size in each mutant verified authentic homologous recombination events, and ruled out the possibility of multicopy insertion of the selection markers. (D) Reverse transcript-PCR confirmed the absence of expression of Bcuve1 and Bcphr1 in the respective mutants. The constitutively expressed gene β-tubulin was used as a reference. [file Image_1.TIF]

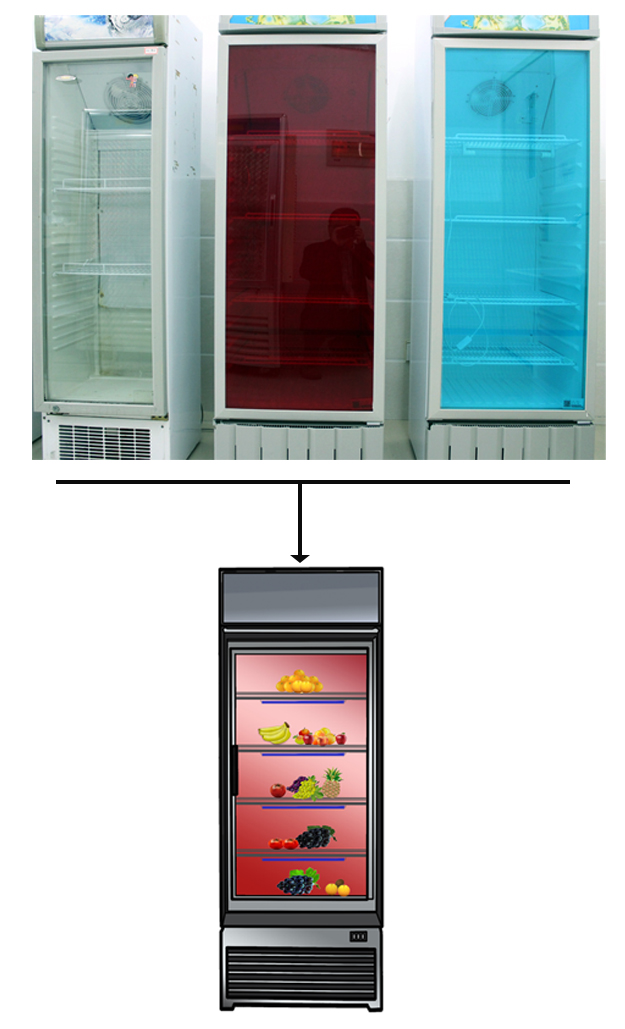

Supplement: FIGURE S2 — Shelf device equipped with UV-C inside and a monochromatic filter on the screen for fresh crop preservation and display. [file Image_2.JPEG]
