# Supplementary material for: Fungal Gene Mutation Analysis Elucidating Photoselective Enhancement of UV-C Disinfection Efficiency Toward Spoilage Agents on Fruit Surface
Source: Front Microbiol. 2018 Jun 12;9:1141. doi: 10.3389/fmicb.2018.01141 (PMC6008522; doi:10.3389/fmicb.2018.01141)
Supplement: TABLE S1 — Primers used in this study. [file Table_1.DOCX]

| **Supplementary table 1**. Primers used in this study | | | | |
| --- | --- | --- | --- | --- |
| NO. | Name | Sequence (5’-3’) | | Purpose |
| P1 | UWCP1 | AAGGTTACCTAAGGCATCAAGA | Amplification of 5’ flank for *Bcwcl1* | |
| P2 | UWCP2 | CCACAGCTGCAGTCTAGAGCAAATACGCCACCACTCAAAG | Amplification of 5’ flank for *Bcwcl1* | |
| P3 | UHY-WCP1 | CTTTGAGTGGTGGCGTATTTGCTCTAGACTGCAGCTGTGG | Amplification of 5’ *Hyg* for *Bcwcl1* deletion | |
| P4 | UHYP2 | CATATGAAATCACGCCATGTAGT | Amplification of 5’ *Hyg* for *Bcwcl1* deletion | |
| P5 | DHYP1 | AAAAGCCTGAACTCACCGC | Amplification of 3’ *Hyg* for *Bcwcl1* deletion | |
| P6 | DHY-WCP2 | CCACATTGACAGCACTCCCCGGGATCCGCTTAGACAA | Amplification of 3’ *Hyg* for *Bcwcl1* deletion | |
| P7 | DWCP1 | TTGTCTAAGCGGATCCCGGGGAGTGCTGTCAATGTGG | Amplification of 3’ flank for *Bcwcl1* | |
| P8 | DWCP2 | GCATTCACCAACGACGCTA | Amplification of 3’ flank for *Bcwcl1* | |
| P9 | *Bcwc*-*1*F | CGACAGTCCTTGGGTGAA | Diagnosis PCR for *Bcwcl1* | |
| P10 | *Bcwc1*-R | GGTTGATACAGATGTGGGT | Diagnosis PCR for *Bcwcl1* | |
| P11 | HJ2-F | CGGCGTAGGGTTGTTCC | Diagnosis PCR for *Hyg* | |
| P12 | HJ2-R | TGGCGACCTCGTATTGG | Diagnosis PCR for Hyg | |
| P13 | WC1-Scr-F | TGGTTTATATTGATGGGTAAAGG | Diagnosis PCR for *Bcwcl1* | |
| P14 | WC1-Scr-R | TGTCAGAACCGTCATCACTCACC | Diagnosis PCR for *Bcwcl1* | |
| P15 | q*β-tubulin-*F | ACCGTTCCAGAGTTGACTCAA | qRT-PCR for *tubulin* reference | |
| P16 | q*β-tubulin-*R | GCAAGAAAGCCTTTCTTCTGA | qRT-PCR for *tubulin* reference | |
| P17 | q*Bcuve1-*F | GCTGCCACGCTCGATCGC | qRT-PCR for *Bcuve1* | |
| P18 | q*Bcuve1-*R | AAGAGCTCCATGATGTCC | qRT-PCR for *Bcuve1* | |
| P19 | q*Bcphr1-*F | TGATCGTCGGCTCTTTTCTC | qRT-PCR for *Bcphr1* | |
| P20 | q*Bcphr1-*R | CATCCCCCGTTATTACTTGC | qRT-PCR for *Bcphr1* | |
| P21 | BCPHR1-up-F | ATAGGGAGGCGTGTACATG | Amplification of 5’ flank for *Bcphr1* | |
| P22 | BCPHR1-up-R | CCACAGCTGCAGTCTAGAGCTTTGAGGATTCGAGAGGTAG | Amplification of 5’ flank for *Bcphr1* | |
| P23 | BCPHR1-middle-F | CTACCTCTCGAATCCTCAAAGCTCTAGACTGCAGCTGTGG | Amplification of *Hyg* for *Bcphr1* deletion | |
| P24 | BCPHR1-middle-R | CATCCCCATCACCATACTCCACCGGGATCCGCTTAGACAA | Amplification of *Hyg* for *Bcphr1* deletion | |
| P25 | BCPHR1-down-F | TTGTCTAAGCGGATCCCGGTGGAGTATGGTGATGGGGATG | Amplification of 3’ flank for *Bcphr1* | |
| P26 | BCPHR1-down-R | AACTTGCCGAAGCGATG | Amplification of 3’ flank for *Bcphr1* | |
| P27 | UVE1-PHR1-up-R | CCGGCCCGAATCGGGAATGCGGCTCTGCTTTGCGAGGAATTGAG | Amplification of *Bcphr1* 5’ flank for double mutation | |
| P28 | UVE1-PHR1-NAT-F | CTCAATTCCTCGCAAAGCAGAGCCGCATTCCCGATTCGGGCCGG | Amplification of *NAT* for double mutation | |
| P29 | UVE1-PHR1-NAT-R | CCCTCTTTATACCTCTTCAAACATCTGTTAGTAATCATCATTAAGTC | Amplification of *NAT* for double mutation | |
| P30 | UVE1-PHR1-down-F | GACTTAATGATGATTACTAACAGATGTTTGAAGAGGTATAAAGAGGG | Amplification of *Bcphr1* 3’ flank for double mutation | |
| P31 | Identi-bcphr1-F | GCCAGTTCGAGTGGATT | Diagnosis PCR for *Bcphr1* | |
| P32 | Identi-bcphr1-R | GAGGGAAGAGGTAGAGTTTAG | Diagnosis PCR for *Bcphr1* | |
| P33 | New-Identi-5’-bcphr1-F | TCCTGTCTGCCACTCCTCG | Diagnosis PCR for *Bcphr1* | |
| P34 | Identi-3’-bcphr1-R | TCTAAATCAGCCACTATGC | Diagnosis PCR for *Bcphr1* | |
| P35 | Identi-bcuve1-F | GACTCCTCAAGCCGTTAC | Diagnosis PCR for *Bcuve1* | |
| P36 | Identi-bcuve1-R | ACTCTGCCTTCGCTAATC | Diagnosis PCR for *Bcuve1* | |
| P37 | Identi-5’-bcuve1-F | TGTTCATTGCTGGTGGGC | Diagnosis PCR for *Bcuve1* | |
| P38 | Identi-3’-bcuve1-R | TTCCGCTCATCCTGTCC | Diagnosis PCR for *Bcuve1* | |
| P39 | Nat-JS-F | CTGGCGGCATTATTGGT | Diagnosis PCR for *NAT* | |
| P40 | Nat-JS-R | AGACGGTGTCGGTGGTG | Diagnosis PCR for *NAT* | |
| P41 | Uve1-BK-F | TCAGAGGAGGACCTGCATATGTTTACTTCCCTCTCATTTCACTTGCG | Clone *Bcuve1* to the vector for yeast two hybrid assay | |
| P42 | Uve1-BK-R | CCGCTGCAGGTCGACGGATCCCTACTCCTCATCCCCATCAACC | Clone *Bcuve1* to the vector for yeast two hybrid assay | |
| P43 | PHR1-AD-F | GTACCAGATTACGCTCATATGTCCACGAGAAAAGCAACCGC | Clone *Bcphr1 t*o the vector for yeast two hybrid assay | |
| P44 | PHR1-AD-R | CAGCTCGAGCTCGATGGATCCCTACGAATTCGCTCTCCCCA | Clone *Bcphr1 t*o the vector f**o**r yeast two hybrid assay | |
| P45 | Uve1-GFP-F | TCACATCACAATCGATCCAACCATGTTTACTTCCCTCTCATTTC | Clone *Bcuve1* to the vector for fusion with *GFP* | |
| P46 | Uve1-GFP-R | TTACCTCACCCTTGGAAACCATCTCCTCATCCCCATCAACCTCAAC | Clone *Bcuve1* to the vector for fusion with *GFP* | |
